# The effect of white grub (*Maladera Verticalis*) larvae feeding on rhizosphere microbial characterization of aerobic rice (*Oryza sativa* L.) in Puer City, Yunnan Province, China

**DOI:** 10.1186/s12866-024-03265-w

**Published:** 2024-04-15

**Authors:** Guang Wang, Zhengfei Li, Baoyun Yang, Huquan Yang, Yujie Zhang, Qingping Zeng, Chaojianping Yan, Yanyan He, Yuejin Peng, Wenqian Wang, Bin Chen, Guangzu Du

**Affiliations:** 1 State Key Laboratory of Conservation and Utilization of Biological Resources of Yunnan, College of Plant Protection, Yunnan Agricultural University, Kunming, 650201 China; 2 School of Agriculture, Yunnan University, Kunming, 650500 China

**Keywords:** Aerobic rice, *Oryza sativa*, White grub, *Maladera Verticalis*, Rhizosphere microorganisms, Physicochemical properties, Enzyme activities

## Abstract

**Background:**

Rhizosphere microorganisms are vital in plants’ growth and development and these beneficial microbes are recruited to the root-zone soil when experiencing various environmental stresses. However, the effect of white grub (*Maladera verticalis*) larvae feeding on the structure and function of rhizosphere microbial communities of aerobic rice (*Oryza sativa* L.) is unclear.

**Results:**

In this study, we compared physicochemical properties, enzyme activities, and microbial communities using 18 samples under healthy and *M. verticalis* larvae-feeding aerobic rice rhizosphere soils at the Yunnan of China. 16 S rRNA and ITS amplicons were sequenced using Illumina high throughput sequencing. *M. verticalis* larvae feeding on aerobic rice can influence rhizosphere soil physicochemical properties and enzyme activities, which also change rhizosphere microbial communities. The healthy and *M. verticalis* larvae-feeding aerobic rice rhizosphere soil microorganisms had distinct genus signatures, such as *possible_genus_04* and *Knoellia* genera in healthy aerobic rice rhizosphere soils and *norank_f__SC − I−84* and *norank_f__Roseiflexaceae* genera in *M. verticalis* larvae-feeding aerobic rice rhizosphere soils. The pathway of the metabolism of terpenoids and polyketides and carbohydrate metabolism in rhizosphere bacteria were significantly decreased after *M. verticalis* larvae feeding. Fungal parasite–wood saprotroph and fungal parasites were significantly decreased after *M. verticalis* larvae feeding, and plant pathogen–wood saprotroph and animal pathogen–undefined saprotroph were increased after larvae feeding. Additionally, the relative abundance of *Bradyrhizobium* and *Talaromyces* genera gradually increased with the elevation of the larvae density. Bacterial and fungal communities significantly correlated with soil physicochemical properties and enzyme activities, respectively.

**Conclusions:**

Based on the results we provide new insight for understanding the adaptation of aerobic rice to *M. verticalis* larvae feeding via regulating the rhizosphere environment, which would allow us to facilitate translation to more effective measures.

**Supplementary Information:**

The online version contains supplementary material available at 10.1186/s12866-024-03265-w.

## Background

Rice (*Oryza sativa* L.) is one of the major staple food crops with respect to area under cultivation and total production. It is widely cultivated in Asia and is significant in ensuring global food security. Rice cultivation requires a huge amount of water [1, 2]. In Asia, approximately 50% of irrigation water is consumed by rice cultivation, which accounts for 24–30% of the withdrawal of the world’s total freshwater [1, 2]. In addition, aerobic rice is a method of rice where seeds are sowed directly in well-tilled and leveled fields [3]. Aerobic rice is developed to tackle the water shortage as a sustainable method to enhance rice production [3]. However, aerobic rice cultivation not only faces overground insects damage, such as yellow stem borer (*Scirpophaga incertulas*), rice leaf folder (*Cnophalocrosis medinalis*), brown planthopper (*Nilaparvata lugens*), white-backed planthopper (*Sogatella turcifera*), and green paddy leafhopper (*Nephotetticx virescens*), but also increases the risk of damage caused by root-feeding insects, such as white grubs [4, 5]. The white grub is a destructive root-feeding insect that feeds on corn [6], potato [7], peanut [8], which may also become one of the pests that harm rice with aerobic cultivation. Such as white grubs were a major production constraint in upland rice production in North-Western Himalayas [9], Madagascar [10], Philippines [11]. The larvae usually damage young seedlings and roots of plants, sometimes resulting in significant yield losses [12, 13] and cost input [11]. However, the damage caused by root-feeding insects is a serious problem in aerobic rice cultivation and will remain so for a long time. Therefore, it is important to investigate the effects of white grub on aerobic rice and microenvironment.

After herbivorous insect’s attack plants, plants rely on direct defense mechanisms and recruit pest antagonists mainly via the release of volatile organic compounds [14, 15]. Accumulating evidence shows that changes in other rhizosphere organisms after root-feeding insects are strongly associated with other rhizosphere organisms [16], such as beneficial plant microbes, antagonists/pathogens of root herbivores, competitors, symbiotic microbes, and detritivores. The root-feeding insect *Elateridae larvae* (wireworms) was reported to change the structure, functional diversity, and tolerance of soil fungi and bacteria [17]. *Holotrichia parallela* larvae (white grub) infestation was reported to change the rhizosphere microbiome of peanuts [18]. In addition, there have been many reports on the dynamic responses of conventional rice rhizosphere microorganisms to biotic and abiotic stresses [19–21], and root-feeding insects feeding on plant root changed rhizosphere microorganisms’ structure and functional diversity. However, the effects of white grub on rhizosphere microorganisms of aerobic rice are not completely understood. Therefore, this study investigates the effects of white grub on aerobic rice rhizosphere microorganisms.

Plants can recruit soil microorganisms into the rhizosphere when experiencing various biotic (insects) and abiotic (heavy metal pollution, droughts, floods, and salinity) stresses [22]. Rhizosphere microorganisms play critical roles in enhancing plant growth and development by promoting nutrient acquisition and assimilation, improving soil texture, and secreting and modulating extracellular molecules [23]. A root-feeding insect infestation can recruit some microorganisms into a plant’s rhizosphere, such as bacterial genera *Pseudomonas*, *Bryobacter*, *Chryseobacterium*, and *Roseiarcus* and fungal species *Candida palmioleophila* and *Coniochaeta fasciculata* [18, 24, 25]. The *Pseudomonas* genus, as a plant-growth-promoting rhizobacteria (PGPR), improves plant growth and health [23]. Notably, rhizosphere microorganisms also promote plant defense against herbivores both above- and belowground by providing feeding deterrence or antibiosis [26]. In addition, *Bryobacter* was positively correlated with the content of bioactive compounds and promoted the biosynthesis and accumulation of 1,8-cineole, cypressene, limonene, and α-terpineol [27]. However, the effects of altered rhizosphere microorganisms on the surrounding soil environment are still unclear.

Therefore, the purpose of this study was to investigate the effects of white grub feeding on soil physicochemical properties, soil enzyme activities, and microbial characterizations of the aerobic rice rhizosphere.

## Materials and methods

### Plants

The rice cultivar (*Oryza sativa* L.), Dianheyou615, was grown at the Puer experimental station, Puer City, Yunnan Province, China. Since January 2022, these experimental sites have been planting Dianheyou615 rice and conducting the same agricultural operations. The rhizosphere soil was collected from three distinct sites (Haguoma, Haozhiba, and Nuoguo) in July 2023.

### Soil collection

The damage of white grubs was investigated during the tillering stage by digging out the root soil. When a white grub found in the field was identified as *Maladera verticalis* larvae, the rhizosphere soil of the aerobic rice plant was collected. The loose soil around the *M. verticalis* larvae-feeding (Mv group) plants and the healthy (CK group) aerobic rice plants was shaken off, and the attached soil was brushed gently and collected [28]. Three biological replicates were maintained for each group, with each replicate collected from 15 plants. Then, the samples were quickly stored in ice boxes and returned to the laboratory. The soil samples were used for soil microbiological analysis, and the other collected material was air-dried for soil properties and enzyme activity for detection analysis. The basic information on sampling location, longitude, insect population density, temperature, humidity, latitude, and altitude are shown in Table 1.

**Table 1:** The basic conditions of the sampling sites

Sampling site	Groups	Larvae density (head/m2)	Altitude/m	Temperature	Humidity	Latitude	Longitude
HGM	CK	0	1580	22.0℃	67.5%	22°45′42″N	99°47′59"E
	Mv	0.48		22.0℃	68.0%		
HZB	CK	0	1490	23.2℃	63.5%	22°44′50"N	99°47′59"E
	Mv	0.67		24.3℃	64.2%		
NG	CK	0	1370	23.4℃	70.0%	22°40′41"N	99°48′40"E
	Mv	0.21		24.2℃	70.2%		

CK, healthy aerobic rice; Mv, *M. verticalis* larvae-feeding aerobic rice; HGM, Haguoma; HZB, Haoziba; and NG, Nuoguo

### Analysis of soil physicochemical properties

A pH meter (FE28, METTLER-TOLEDO, USA) was used to determine soil pH in a soil–water suspension (1:2.5, air-dried soil/distilled water for removing CO_2_) after shaking for 30 min. Soil physiochemical properties were measured using the following methods: organic matter—potassium dichromate oxidation spectrophotometric method; total nitrogen—sulfuric acid digestion method + Kjeldahl method; total phosphorus—alkali fusion-Mo-Sb anti spectrophotometric method; available phosphorus—sodium hydrogen carbonate solution-Mo-Sb anti spectrophotometric method; total potassium—NaOH digestion method + flame photometric method; available potassium—ammonium acetate extraction + flame photometric method; and NH_4_-N—KCl_2_ extraction + indophenol blue spectrophotometric method.

### Determination of soil enzyme activities

Urease activity, acid phosphatase activity, catalase activity, β-glucosidase, polyphenol oxidase, and acid invertase in rhizosphere soil were determined using commercially available kits (MolFarming, Nanjing, China). Briefly, fresh soil samples were naturally air-dried and sieved through a 30–50 mesh sieve.

#### Urease activity

(1) Add 100 µL of toluene to 0.1 g of soil and let sit at room temperature for 15 min. (2) Add 500 µL of reagent I and 1,000 µL of reagent II to (1) the sample. Then, incubate the mixture at 37℃ for 24 h and centrifuge at 10,000 rpm for 10 min. Add 80 µL of reagent III and 60 µL of reagent IV to 200 µL of (2) the supernatant. Then, incubate the mixture at room temperature for 20 min and add 660 µL of distilled water. The absorbance value was measured at 630 nm.

#### Acid phosphatase activity

(1) Add 50 µL of toluene to 0.1 g of soil and let sit at room temperature for 15 min. (2) Add 250 µL of reagent I and 250 µL of reagent II to (1) the sample. Then, incubate the mixture at 37℃ for 24 h. (3) Add reagent III (500 µL) to (2) the sample and centrifuge at 10,000 rpm for 10 min. (4) Add 700 µL of reagent IV, 100 µL of reagent V, and 100 µL of reagent VI to 100 µL of (3) the supernatant and allow a standing reaction time of 10 min. The absorbance value was measured at 630 nm.

#### Catalase activity

(1) Add 1,000 µL of reagent I to 0.05 g of soil and incubate for 30 min. Then, add 200 µL of reagent II and centrifuge at 10,000 rpm for 10 min. (2) Mix 100 µL of (1) the supernatant and 1,000 µL of reagent III and allow the mixture to stand for 5 min. The absorbance value was measured at 405 nm.

#### β-glucosidase

(1) Add 50 µL of toluene to 0.1 g of soil and allow the mixture to sit at room temperature for 15 min. (2) Add 500 µL of reagent I and 100 µL of reagent II to (1) the sample. Then, incubate at 37℃ for 3 h and then boil for 5 min. (3) Add 900 µL of reagent IV to 100 µL of (2) the supernatant and allow the mixture to stand for 15 min. The absorbance value was measured at 400 nm.

#### Polyphenol oxidase

(1) Add 500 µL of reagent I to 0.1 g of soil and incubate at 30℃ for 2 h. Add 200 µL of reagent II and 2,000 µL of ether and allow the mixture to stand at room temperature for 30 min. The absorbance value was measured at 430 nm.

#### Acid invertase

(1) Add 50 µL of toluene to 0.1 g of soil and allow the mixture to stand at room temperature for 15 min. (2) Add 250 µL of reagent I and 750 µL of reagent II to (1) the sample and incubate at 37℃ for 24 h. Then, centrifuge at 10,000 rpm for 10 min. (2) Add 300 µL of reagent III to (1) the supernatant and then boil for 5 min. (3) Add 1,000 µL of distilled water to (2) the sample. The absorbance value was measured at 540 nm.

### Determine bacterial and fungal densities

The rhizosphere soil (10 g) was suspended in 90 mL of sterile saline (10% w/v) and agitated at 150 rpm for 30 min. The suspension was diluted serially to 10^-1^-10^-6^. Aliquots of 100 µL of suspension (10^-4^-10^-6^) were spread on Luria–Bertani (LB) plates to cultivate bacteria and 100 µL of suspension (10^-1^-10^-3^) was spread on Thayer–Martin medium (hopebio, Qingdao, China) with rose Bengal and 0.003% streptomycin sulfate on plates to cultivate fungi. The plates were incubated at 25 ± 1℃ for 3 days. After incubation, the colony-forming units (CFU/g) of bacteria and fungi were counted.

### DNA extraction and MiSeq sequencing

Rhizosphere microorganism genomic DNA isolation, 16 S rDNA, and ITS gene sequencing were completed with the help of Majorbio Co. Ltd., Shanghai. Isolation of microbial DNA from the rhizosphere soil was performed using a Qiagen E. Z.N. A.® Soil DNA Kit (Omega Bio-Tek, USA). The 16 S rRNA-encoding gene and ITS rDNA were amplified from extracted DNA using bacterial primers, 338 F/806R [29], and fungal primers, ITS1F/ITS2R, respectively [30]. The PCR product was identified via 2% gel electrophoresis, purified using AxyPrep DNA Gel Extraction Kit (AXYGEN, Union, CA), and quantified via QuantiFluor™-ST (Promega, Wisconsin, USA). The pooled amplicon library was then sequenced on the Illumina MiSeq platform using the TruSeqTM DNA Sample Prep Kit (Illumina, USA) according to the manufacturer’s instructions. Flash and Trimmomatic programs were used to screen and trim the raw sequences, including quality trimming, chimera detection, and removal. The Silva database (Release 138) was used to align the sequences for 16 S rRNA gene data [31], and the Unite database (version 8.0) was used to align ITS gene data [32]. The operational taxonomic unit (OTU) was aligned using the Silva and Unite databases to obtain species taxonomic information for each OTU. The OTU table was manually filtered, i.e., chloroplast sequences in all samples were removed.

#### Bacterial and fungal functional analyses

The software Tax4Fun (Version 0.3.1) and FUNGuild (Version 1.0) were used for the functional prediction of 16 S and ITS amplicon sequencing results, respectively. Tax4Fun was used to obtain three levels of metabolic pathway information and pathway abundance [33]. FUNGuild was used to obtain fungal functional guild data [34].

### Statistical analysis

The alpha diversity index of Shannon–Wiener was calculated using Mothur (Version 1.30.1). The number of reads of species was also used for principal component analysis (PCA) based on the Euclidean distance using Qiime (Version 1.9.1). Species with significant differences in sample classification were identified using linear discriminant analysis (LDA ≥ 2). The correlation analyses were conducted using R software (Version 4.1.1) and visualized using the pheatmap package in R software. An unpaired, two-sided Student’s t-test was used when two groups were compared. Differences were considered to be statistically significant if *P* < 0.05.

## Results

### *M. verticalis* larvae feeding influenced soil physicochemical properties

**Fig. 1:**
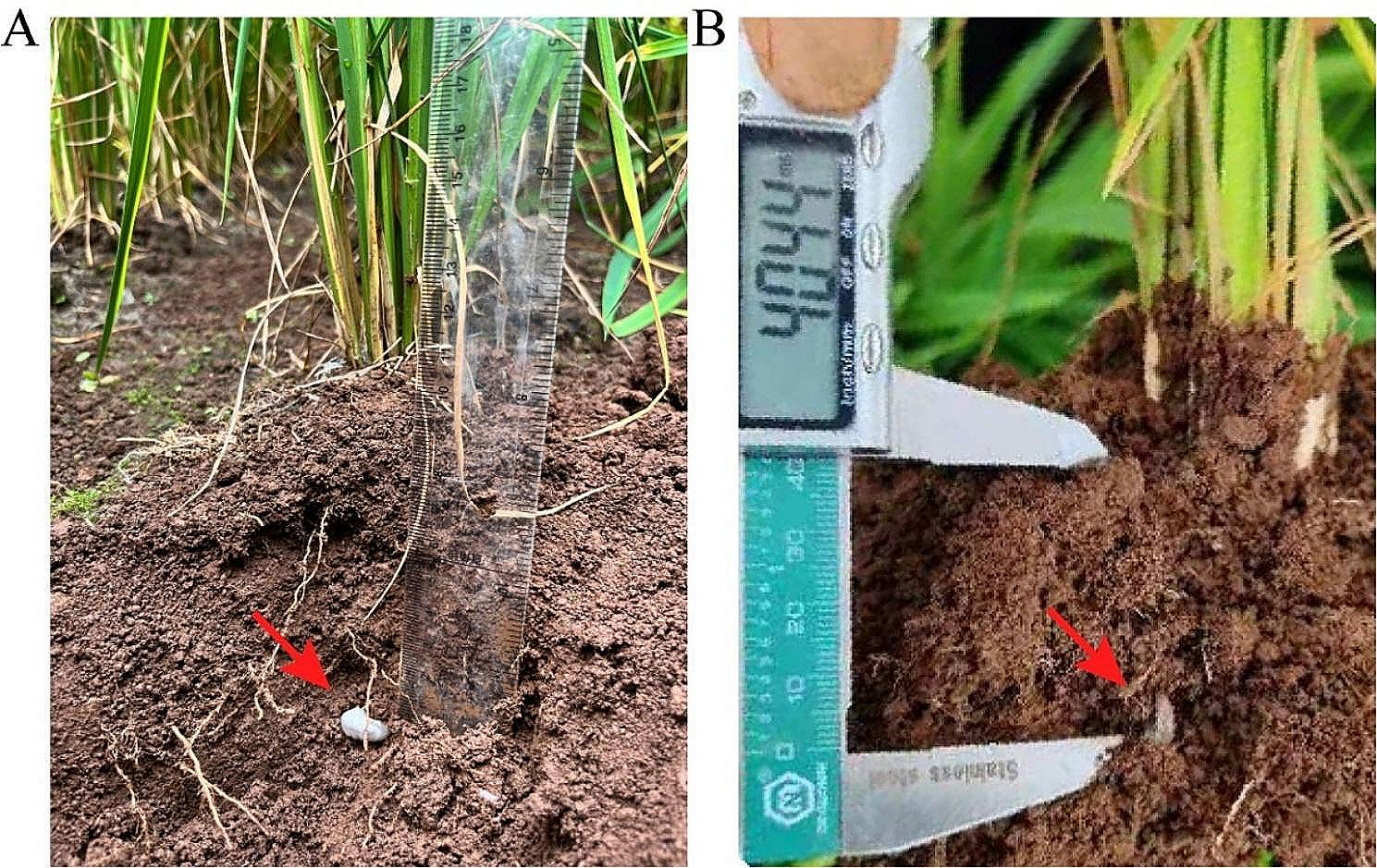
An image of a white grub (*Maladera verticalis* larvae) in the field, which harms aerobic rice roots

The irrigated rice Dianheyou 615 can be grown in mountainous areas in the Yunnan-Guizhou plateau in China, where below 1900 m above sea level and the rainfall exceeds 1000 millimeters. Aerobic rice cultivation model will increase the risk of damage caused by *M. verticalis* larvae (Fig. 1). Next, we examined the changes in the physicochemical properties of rhizosphere soil after the harm of *M. verticalis* larvae. As shown in Table 2, M. *verticalis* larvae feeding on aerobic rice plants significantly increased the pH, organic matter, total K, and available K contents of the HGM-Mv group and significantly decreased the total N content compared with the HGM-CK group. *M. verticalis* larvae feeding significantly raised the pH of the HZB-Mv group, while *M. verticalis* larvae feeding significantly reduced the total N, total P, available P, and available K contents compared with the HZB-CK group. In addition, the total P, available P, and available K contents were markedly elevated in the NG-Mv group compared with the NG-CK group, and the pH and organic matter contents were inhibited. However, the content of NH4+-N was not affected by *M. verticalis* larvae feeding in the three regions.

**Table 2:** Physicochemical characteristics of healthy and *M. verticalis* larvae-feeding aerobic rice rhizosphere soils within different regions

Plot		HGM			HZB			NG	
Groups		CK	Mv		CK	Mv		CK	Mv
pH		4.82 ± 0.04	5.05 ± 0.03^**^		5.58 ± 0.02	5.82 ± 0.01^**^		4.86 ± 0.02	4.71 ± 0.01^**^
Organic matter/(g/kg)		44.70 ± 0.34	52.30 ± 0.77^**^		48.28 ± 0.3	48.34 ± 0.1		40.46 ± 0.3	37.08 ± 0.48^**^
Total N/(g/kg)		2.33 ± 0.02	2.20 ± 0.02^**^		2.56 ± 0.02	2.50 ± 0.01^**^		2.22 ± 0.08	2.33 ± 0.02
Total P/(g/kg)		2.07 ± 0.02	2.02 ± 0.02		3.26 ± 0.01	2.90 ± 0.02^**^		1.53 ± 0.02	1.62 ± 0.02^*^
Available P/(mg/kg)		6.37 ± 0.45	5.72 ± 0.19		30.28 ± 0.53	8.43 ± 0.07^**^		10.38 ± 0.33	16.29 ± 0.09^**^
Total K/(g/kg)		7.77 ± 0.08	9.20 ± 0.02^**^		11.34 ± 0.09	11.69 ± 0.2		14.54 ± 0.2	14.28 ± 0.14
Available K/(mg/kg)		135.37 ± 0.78	145.4 ± 0.8^**^		520.93 ± 4.96	379.32 ± 2.47^**^		169.58 ± 1.12	203.02 ± 0.79^**^
NH4-N/(mg/kg)		5.48 ± 0.45	5.88 ± 0.16		6.46 ± 0.51	6.65 ± 0.47		5.66 ± 0.44	5.79 ± 0.65

The error bars represent the standard errors. ^*^P < 0.05 and ^**^P < 0.01 vs. the CK

CK, healthy aerobic rice; Mv, *M. verticalis* larvae-feeding aerobic rice; HGM, Haguoma; HZB, Haoziba; and NG, Nuoguo

### *M. Verticalis* larvae feeding influenced soil enzyme activities

As shown in Table 3, M. *verticalis* larvae feeding significantly increased the activity of urease, phosphatase, β-glucosidase, polyphenol oxidase, and acid invertase in the HGM samples. In the HZB samples, *M. verticalis* larvae feeding significantly increased urease, catalase, and β-glucosidase activities and significantly decreased phosphatase and acid invertase activities. *M. verticalis* larvae feeding markedly decreased urease and phosphatase activities in the NG samples.

**Table 3:** Soil enzyme activities of healthy and *M. verticalis* larvae-feeding aerobic rice rhizosphere soils within different regions

Plot		HGM			HZB			NG	
Groups		CK	Mv		CK	Mv		CK	Mv
Urease (µg/g·24 h)		643.41 ± 16.63	736.18 ± 9.04^**^		591.41 ± 3.26	719.73 ± 8.66^**^		1102.52 ± 33.93	1005.64 ± 11.66^*^
Phosphatase (mg/g·24 h)		3.22 ± 0.07	3.54 ± 0.08^*^		2.62 ± 0.07	2.42 ± 0.07^*^		4.2 ± 0.04	3.90 ± 0.03^**^
Catalase (µmol/g·h)		1907.90 ± 84.88	1877.12 ± 39.01		1763.77 ± 38.66	2124.78 ± 33.50^**^		1146.42 ± 30.52	1131.93 ± 33.78
β-glucosidase (µg/g·h)		72.32 ± 0.48	80.04 ± 3.3^*^		60.71 ± 1.84	64.95 ± 0.96^*^		114.24 ± 1.38	118.36 ± 2.2
Polyphenol oxidase (mg/g·24 h)		2.72 ± 0.24	4.40 ± 0.12^**^		3.94 ± 0.37	4.16 ± 0.54		4.27 ± 0.38	4.04 ± 0.44
Acid invertase (mg/g·24 h)		8.91 ± 0.26	10.63 ± 0.28^**^		8.50 ± 0.19	7.42 ± 0.14^**^		12.05 ± 0.26	12.22 ± 0.21

The error bars represent the standard errors. ^*^P < 0.05 and ^**^P < 0.01 vs. the CK

CK, healthy aerobic rice; Mv, *M. verticalis* larvae-feeding aerobic rice; HGM, Haguoma; HZB, Haoziba; and NG, Nuoguo

### Sequencing data statistics

The OTU number of bacteria in each sample was at least 24.32 (Table S1). The coverage of bacteria in all samples were > 98%. The rarefaction curves of these sequences tend to be flat in bacteria, indicating a sufficient depth for the sequencing depth. In addition, the OTU number of fungi in each sample was at least 672. The coverage of bacteria in all samples were > 99%. The rarefaction curves of these sequences tend to be flat in fungi, indicating a sufficient depth for the sequencing (Figure F1).

### The diversity and composition of rhizosphere bacteria after *M. verticalis* larvae feeding

**Fig. 2:**
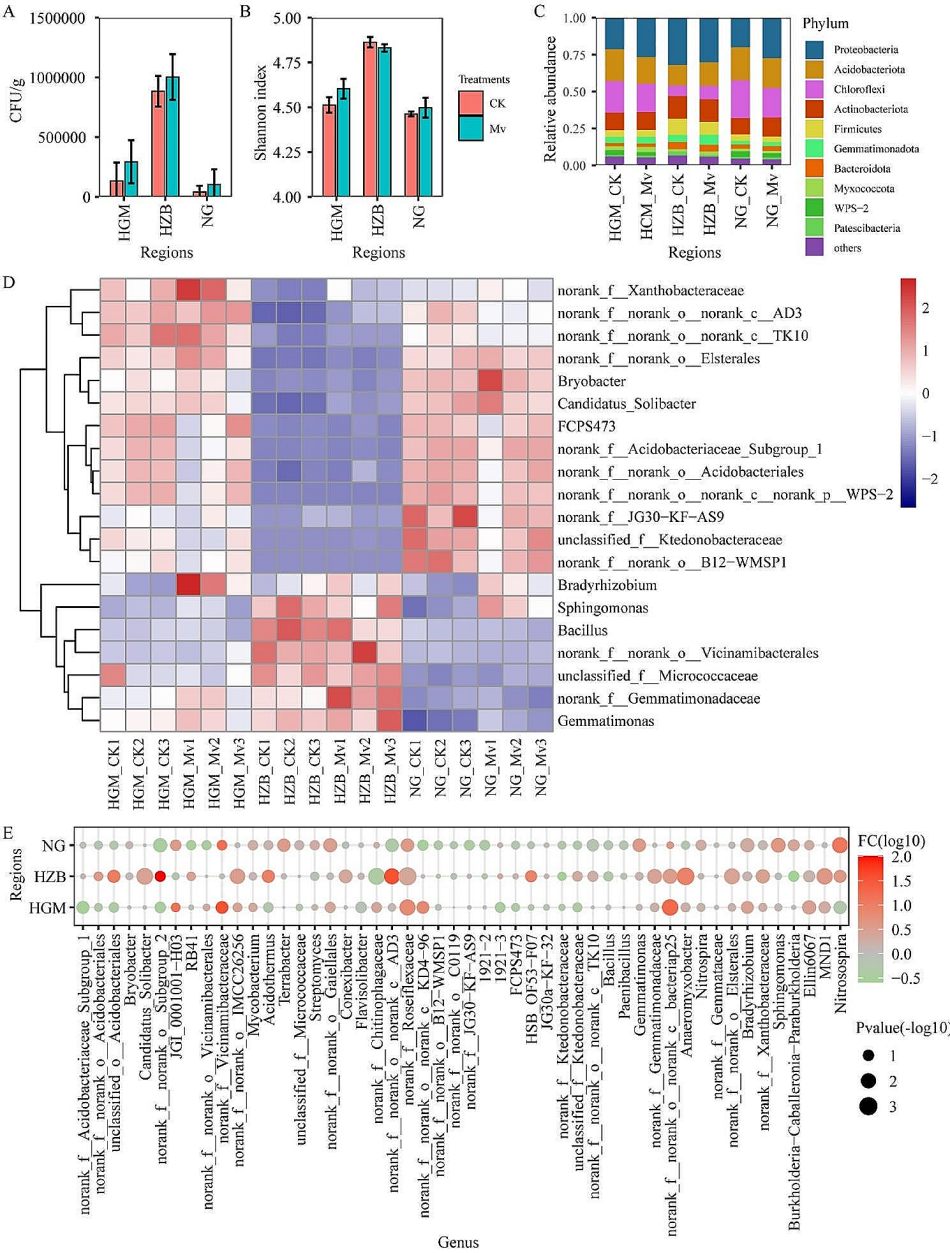
The diversity and composition of rhizosphere bacteria after *M. verticalis* larvae feeding. (**A**) The colony-forming units of bacteria in the LB medium. (**B**) Alpha diversity of the Shannon index at the bacterial genus level. (**C**) Phylum-level relative frequency in healthy and *M. verticalis* larvae-feeding aerobic rice rhizosphere bacteria. (**D**) Heatmap of genus-level in healthy and *M. verticalis* larvae-feeding aerobic rice rhizosphere bacteria. (**E**) Genus-level relative frequency in healthy and *M. verticalis* larvae-feeding aerobic rice rhizosphere bacteria. The relative frequency of log_10_ fold change (log_10_FC) was included in the plot. CK, healthy aerobic rice; Mv, *M. verticalis* larvae-feeding aerobic rice; HGM, Haguoma; HZB, Haoziba; and NG, Nuoguo

**Fig. 3:**
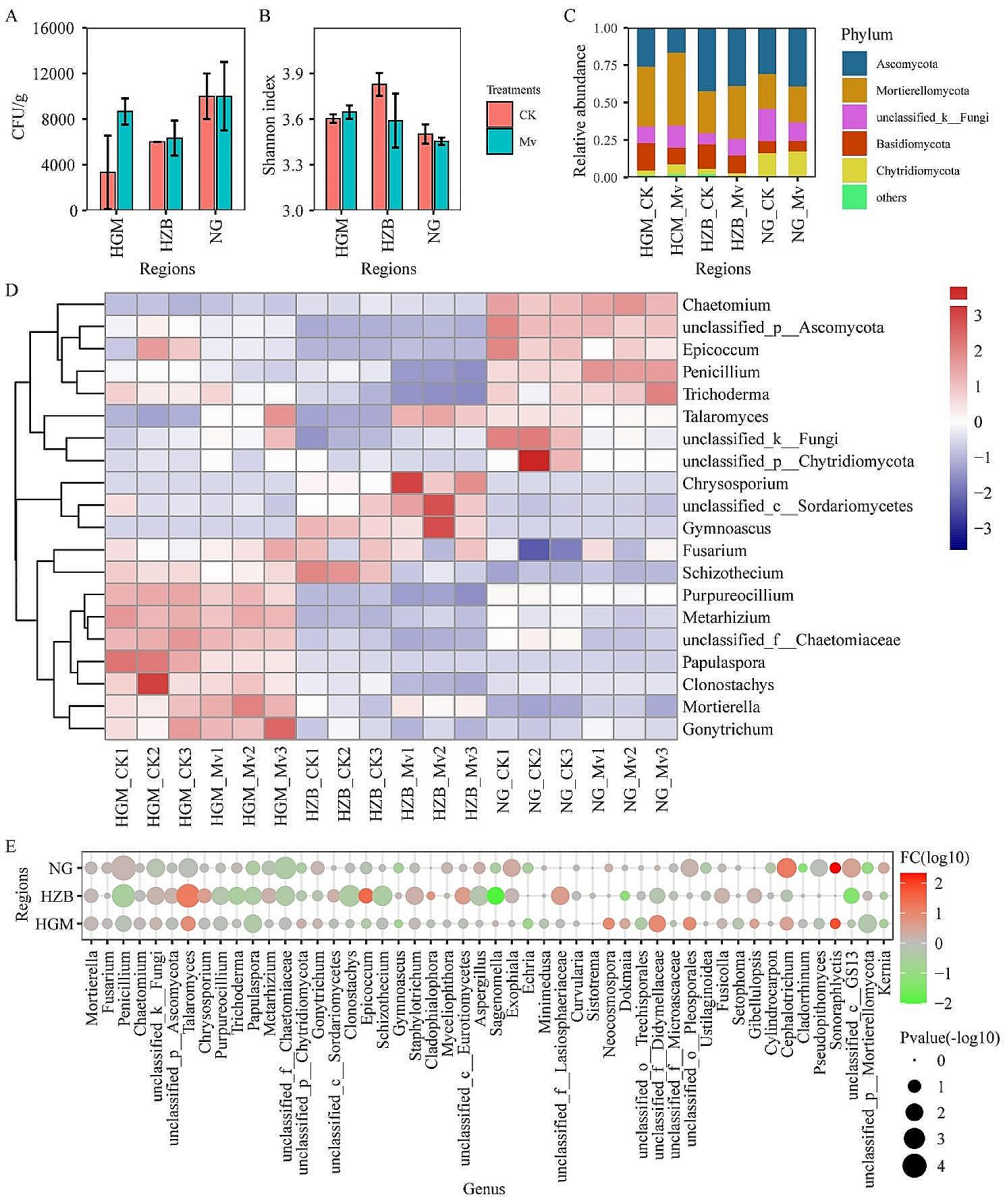
The diversity and composition of rhizosphere fungi after *M. verticalis* larvae feeding. (**A**) The colony-forming units of fungi in Thayer–Martin medium with rose bengal and 0.003% streptomycin sulfate. (**B**) Alpha diversity of Shannon index at the fungal genus level. (**C**) Phylum-level relative frequency in healthy and *M. verticalis* larvae-feeding aerobic rice rhizosphere fungi. (**D**) Heatmap of genus-level in healthy and *M. verticalis* larvae-feeding aerobic rice rhizosphere fungi. (**E**) Genus-level relative frequency in healthy and *M. verticalis* larvae-feeding aerobic rice rhizosphere fungi. The relative frequency of log_10_ fold change (log_10_FC) was included in the plot. CK, healthy aerobic rice; Mv, *M. verticalis* larvae-feeding aerobic rice; HGM, Haguoma; HZB, Haoziba; and NG, Nuoguo

We next explored the effects of *M. verticalis* larvae feeding on aerobic rice rhizosphere bacteria. *M. verticalis* larvae feeding increased bacterial density in the three regions compared with the CK group (Fig. 2A). PCA was used to determine whether there were significant differences in the bacterial composition of *M. verticalis* larvae-feeding aerobic rice soil samples between the three regions. As shown in Figure S2, the samples were divided into three groups by region (Figure S2A). These samples were divided into two groups according to the Mv and CK groups in the HGM (Figure S2B), HZB (Figure S2C), and NG (Figure S2D) regions. The results suggest that *M. verticalis* larvae feeding was the main factor leading to differences in rhizosphere bacteria in a plot. However, the Shannon index (Fig. 2B) and the relative abundance of the top 10 phyla (Fig. 2C) showed no significant differences in the Mv and CK groups in same experimental area. In addition, the top 20 genera were displayed using a heatmap. The 9 healthy and 9 *M. verticalis* larvae-feeding aerobic rice rhizosphere bacteria formed three distinct groups with three regions (Fig. 3D). *Bradyrhizobim* was elevated in the *M. verticalis* larvae-feeding aerobic rice rhizosphere soils. Then, the top 50 bacterial genera that distinguish the *M. verticalis* larvae-feeding aerobic rice rhizosphere bacteria and the healthy aerobic rice rhizosphere bacteria were recorded by log_10_FC levels. The relative abundances of 26, 37, and 24 genera were increased after *M. verticalis* larvae feeding in the HGM-Mv, HZB-Mv, and NG-Mv groups, respectively (Fig. 2D, Table S2). On the contrary, *M. verticalis* larvae feeding decreased the relative abundances of 24, 13, and 26 genera in the samples from the HGM, HZB, and NG sites (Fig. 2D, Table S2).

### The diversity and composition of rhizosphere fungi after *M. Verticalis* larvae feeding

*M. verticalis* larvae feeding did not significantly change fungal density in the rhizosphere soil of three regions (Fig. 3A). PCA was used to determine whether there were significant differences in the fungal composition of *M. verticalis* larvae-feeding soil samples between the tested regions. As shown in Figure S2, the samples were divided into three groups by region (Figure S3A). These samples were then divided into two groups according to the Mv and CK groups in the HGM (Figure S3B), HZB (Figure S3C), and NG (Figure S3D) regions. The results suggest that *M. verticalis* larvae feeding was the main factor leading to differences in rhizosphere fungi in a plot. However, *M. verticalis* larvae feeding did not significantly change the Shannon index (Fig. 3B) and relative abundance of the top five phyla (Fig. 3C) in same experimental area. In addition, the top 20 genera were displayed using a heatmap. The 9 healthy and 9 *M. verticalis* larvae-feeding aerobic rice rhizosphere bacteria formed three distinct groups with three regions (Fig. 3D). *Papulaspora* was decreased in the *M. verticalis* larvae-feeding aerobic rice rhizosphere soils. Then, the top 50 fungal genera that distinguish the Mv and CK were recorded by log_10_FC. The relative abundances of 19, 24, and 24 genera were increased in the HGM-Mv, HZB-Mv, and NG-Mv groups, respectively (Fig. 3D, Table S3). On the contrary, *M. verticalis* larvae feeding decreased the relative abundances of 27, 25, and 21 genera in the HGM, HZB, and NG samples (Fig. 3D, Table S3).

### LDA revealed the most featured microbial genera in healthy and *M. verticalis* larvae-feeding aerobic rice rhizosphere microorganisms

**Fig. 4:**
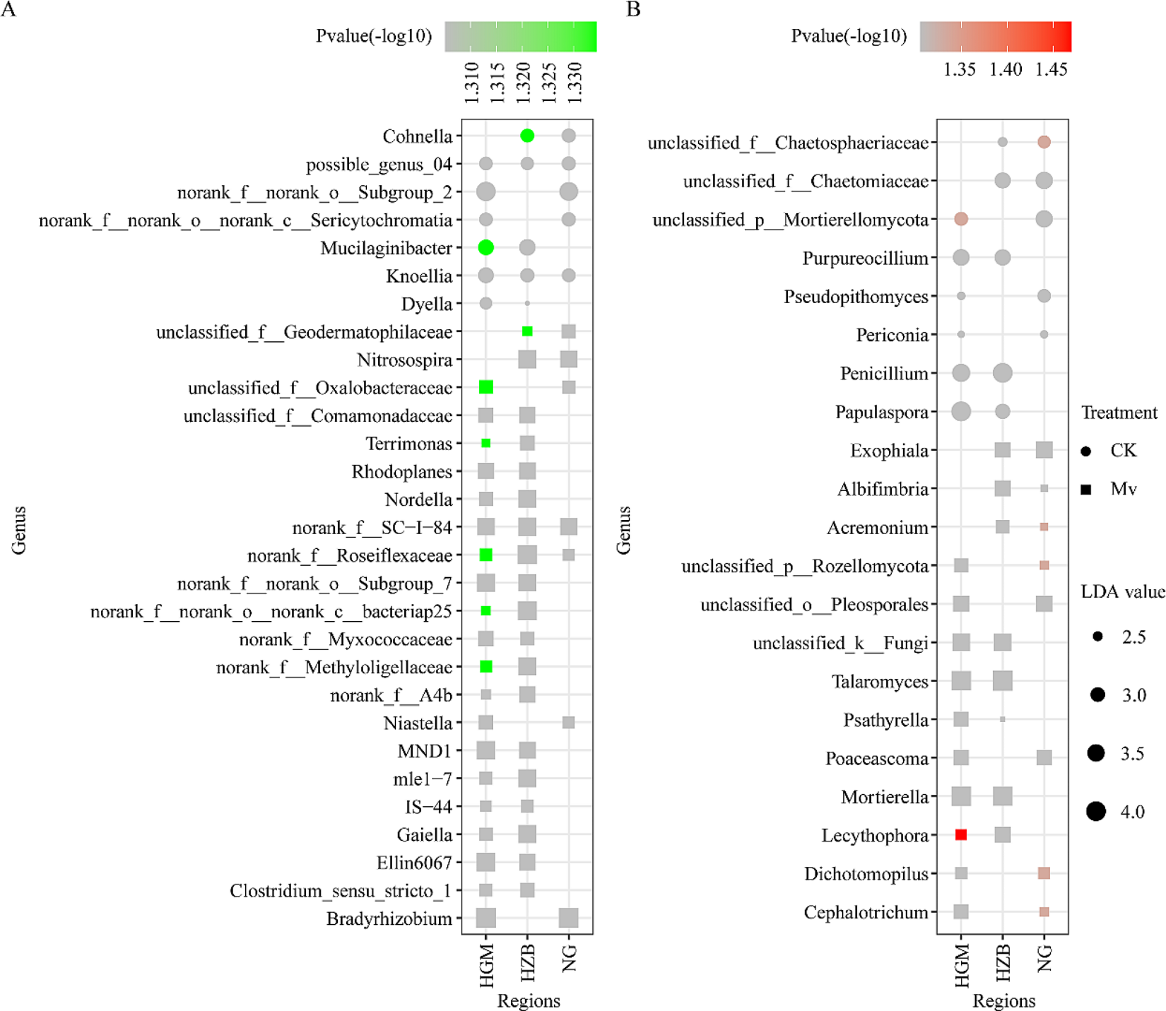
Significant differences in genus abundance that discriminate healthy and *M. verticalis* larvae-feeding aerobic rice rhizosphere microorganisms. LDA (LDA value > 2.0 and P value ≤ 0.05) scores of top featured bacterial (**A**) and fungal (**B**) genera in healthy and *M. verticalis* larvae-feeding aerobic rice rhizosphere. The square and circle represent the CK and Mv groups, respectively. CK, healthy aerobic rice; Mv, *M. verticalis* larvae-feeding aerobic rice; HGM, Haguoma; HZB, Haoziba; and NG, Nuoguo

To identify the featured genus associated with *M. verticalis* larvae feeding, we performed LDA on microbial abundance profiles at the genus level. After comprehensive analysis and screening with the Venn diagram tool (Figure S4), at the bacterial level, seven featured genera were identified in the healthy aerobic rice rhizosphere (Figure S4A), five genera were shared among the two regions, and *possible_genus_04* and *Knoellia* genera were shared among the three regions (Fig. 4A). *M. verticalis* larvae-feeding aerobic rice rhizosphere bacteria featured 22 genera (Figure S4B), 20 genera were shared among the two regions, and *norank_f_SC-I-84* and *norank_f_Roseiflexaceae* genera were shared among the three regions (Fig. 4A). At the fungal level, 8 and 13 featured genera were identified in the healthy and *M. verticalis* larvae-feeding aerobic rice rhizosphere, respectively (Figure S4C and S4D), but no featured genera were shared among the three regions (Fig. 4B). Our results further confirm that the healthy and *M. verticalis* larvae-feeding aerobic rice rhizosphere microorganisms have distinct taxonomical signatures.

### *M. verticalis* larvae-feeding induced changed aerobic rice rhizosphere soil microbial community and composition

**Fig. 5:**
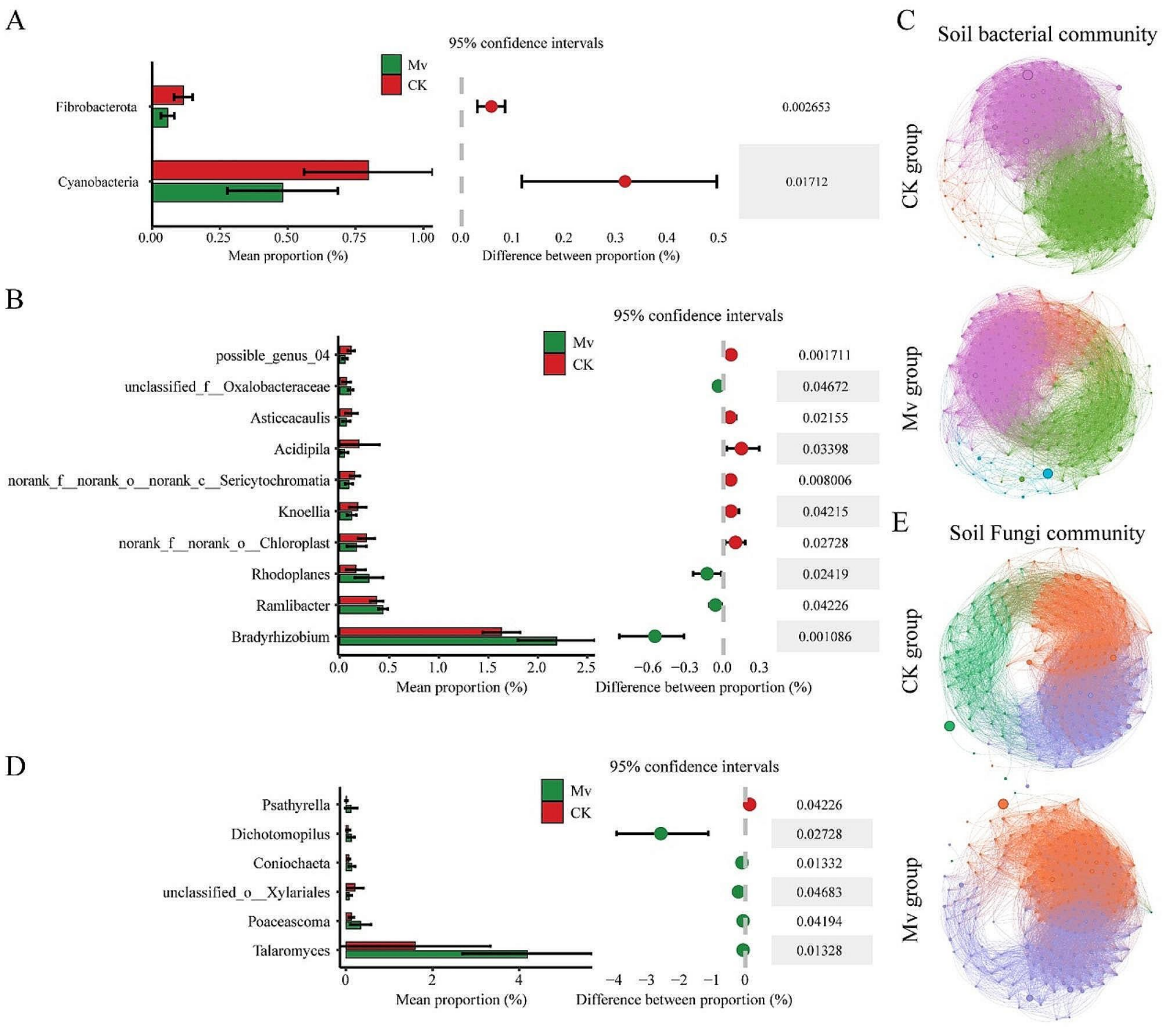
*M. verticalis* larvae-feeding induced changed aerobic rice rhizosphere soil microbial community and composition. Histogram showing significantly different phyla (**A**) and genera (**B**). (**C**) Aerobic rice rhizosphere soil bacterial community. (**D**) Histogram showing significantly different genera. (**E**) Aerobic rice rhizosphere soil fungi community. CK, healthy aerobic rice; Mv, *M. verticalis* larvae-feeding aerobic rice

To further investigate the effects of *M. verticalis* larvae feeding on aerobic rice rhizosphere soil microorganisms, we merged all CK and Mv groups, separately. In the bacterial level, *M. verticalis* larvae-feeding significantly reduced the relative abundances of Fibrobacterota and Cyanobacteria phyla (Fig. 5A). With respect to soil bacterial composition, *M. verticalis* larvae-feeding significantly increased the genera of *Bradyrhizobium*, *Ramlibacter*, *Rhodoplanes*, and *unclassified_f__Oxalobacteraceae*, but decreased the genera of *norank_f__norank_o__Chloroplast*, *Knoellia*, *norank_f__norank_o__norank_c__Sericytochromatia*, *Acidipila*, *Asticcacaulis*, and *possible_genus_04* (Fig. 5B). Besides, *M. verticalis* larvae-feeding changed aerobic rice rhizosphere soil bacterial community (Fig. 5C). The network was assigned to 4 modules with healthy and *M. verticalis* larvae-feeding aerobic rice rhizosphere soils. The modularity decreased from 0.355 in healthy aerobic rice rhizosphere soils to 0.277 in *M. verticalis* larvae-feeding aerobic rice rhizosphere soils (Table S4). Modules I, III, and IV displayed more proportion in the Mv group (49%, 12.5%, and 6%) than CK group (48.5%, 5.55%, and 1%) (Fig. 5C and Table S6). However, the module I was decreased from 45% in healthy aerobic rice rhizosphere soils to 32.5% in *M. verticalis* larvae-feeding aerobic rice rhizosphere soils. The network was assigned to 11 and 12 phyla within healthy and *M. verticalis* larvae-feeding aerobic rice rhizosphere soils, respectively. Among them, nodes in the CK network were mostly from Acidobacteriota (25%), Proteobacteria (22.55%), and Chloroflexi (22%) and in the Mv network mostly from Proteobacteria (25%), Acidobacteriota (22.5%), and Chloroflexi (20%) (Table S5).

In the fungi level, *M. verticalis* larvae feeding did not significantly change any soil fungal phyla in the aerobic rice rhizosphere soils. *M. verticalis* larvae-feeding significantly increased the genera of *Talaromyces*, *Poaceascoma*, *unclassified_o__Xylariales*, *Coniochaeta*, and *Dichotomopilus* but decreased the genus of *Psathyrella* (Fig. 5D). Besides, *M. verticalis* larvae-feeding changed aerobic rice rhizosphere soil fungal community (Fig. 5E). The network was assigned to 3 modules with healthy and *M. verticalis* larvae-feeding aerobic rice rhizosphere soils. The modularity increased from 0.207 in healthy aerobic rice rhizosphere soils to 0.255 in *M. verticalis* larvae-feeding aerobic rice rhizosphere soils (Table S4). Modules I and II displayed more proportion in the Mv group (54% and 44.67%) than CK group (40.44% and 35.9%) (Fig. 5E and Table S4). However, the module III was decreased from 23.66% in healthy aerobic rice rhizosphere soils to 1.02% in *M. verticalis* larvae-feeding aerobic rice rhizosphere soils. The network was assigned to 6 phyla within healthy and *M. verticalis* larvae-feeding aerobic rice rhizosphere soils. Among them, nodes in the CK network were mostly from Ascomycota (80.81%) and in the Mv network mostly from Ascomycota (77.16%) (Table S5). Notably, the change of bacterial community and composition were greater than fungal community and composition with *M. verticalis* larvae-feeding stress.

### Rhizosphere microbial community functional prediction

**Fig. 6:**
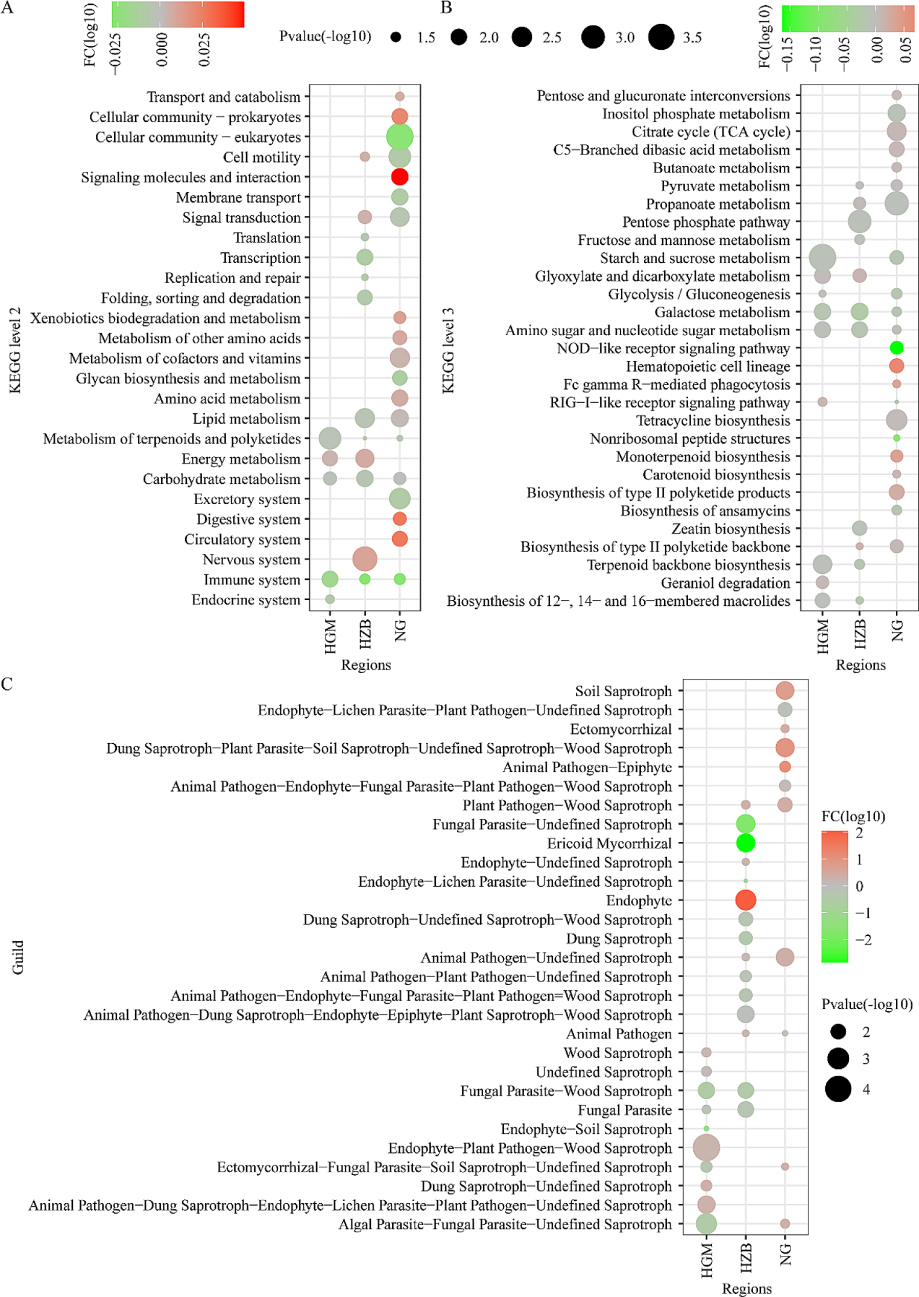
Rhizosphere microbial community functional prediction. (A) Statistical differences in predicated bacterial functional characteristics between CK and Mv rhizosphere soil at KEGG level 2 (**A**) and KEGG level 3 (**B**). (**C**) Statistical differences in predicated fungal functional characteristics between CK and Mv rhizosphere soil. The relative abundance of log_10_ fold change (log_10_FC) was included in the plot. CK, healthy aerobic rice; Mv, *M. verticalis* larvae-feeding aerobic rice; HGM, Haguoma; HZB, Haoziba; and NG, Nuoguo

The bacterial communities’ functional prediction results showed (Fig. 6A, Table S6) that at level 2, we identified 1 (HGM), 4 (HZB), and 10 (NG) pathways significantly enriched in the *M. verticalis* larvae-feeding aerobic rice rhizosphere bacteria, while 4 (HGM), 8 (HZB), and 9 (NG) pathways were significantly depleted. The pathways of the metabolism of terpenoids and polyketides, carbohydrate metabolism, and immune system were shared among the three regions, and they were significantly more abundant in the healthy aerobic rice rhizosphere. To further study the functional information of rhizosphere bacteria, the metabolism of terpenoids and polyketides, carbohydrate metabolism, and the immune system of each experimental group were compared at KEGG level 3. As shown in Fig. 6B and Table S7, we detected 3 (HGM), 3 (HZB), and 12 (NG) pathways significantly enriched in the *M. verticalis* larvae-feeding aerobic rice rhizosphere bacteria, and 6 (HGM), 8 (HZB), and 9 (NG) pathways significantly enriched in the healthy aerobic rice rhizosphere bacteria. Compared with the healthy aerobic rice rhizosphere, *M. verticalis* larvae feeding significantly depleted the enrichment of galactose metabolism and amino sugar and nucleotide sugar metabolism in the three regions.

The fungal communities’ functional prediction results showed that 10 (HGM), 15 (HZB), and 11 (NG) different ecological guilds were present among the communities of the aerobic rice rhizospheres, in addition to unidentified guilds (Fig. 6C, Table S8). Fungal parasite–wood saprotroph and fungal parasite abundance at the HGM and HZB regions were significantly decreased with *M. verticalis* larvae feeding. In addition, the abundance of plant pathogen–wood saprotroph (a type of fungi that obtains nutrients by damaging host plant cells and degrading wood cells to obtain nutrients) and animal pathogen–undefined saprotroph (a type of fungi that obtains nutrients by damaging host animal cells and degrading unknown substances to obtain nutrients) in samples from the HZB and NG regions notably increased after *M. verticalis* larvae feeding.

### Relationships between soil properties and rhizosphere microbial community structures

**Fig. 7:**
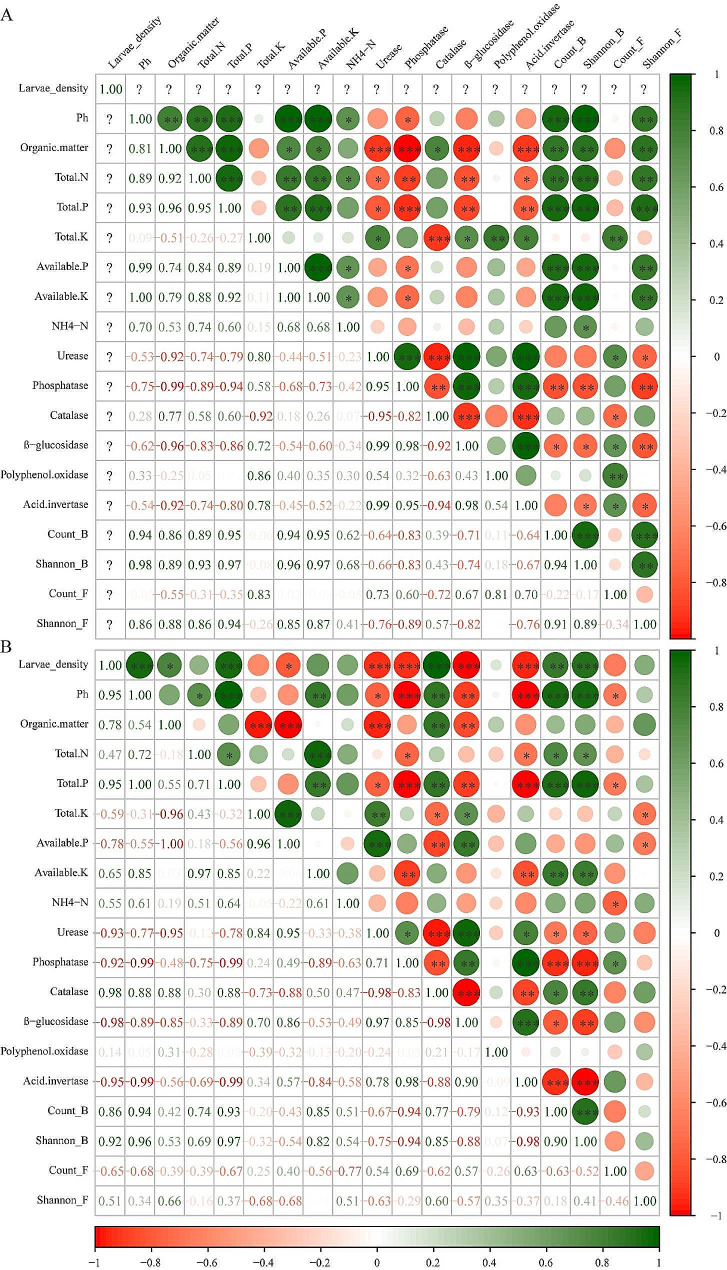
Relationship between soil properties and rhizosphere microbial community structures. (A) Relationship between healthy aerobic rice rhizosphere soils properties and rhizosphere microbial community structures. (B) Relationship between *M. verticalis* larvae-feeding aerobic rice rhizosphere soils properties and rhizosphere microbial community structures. ^*^, ^**^, and ^***^ indicate significant differences at *P* < 0.05, *P* < 0.01, and *P* < 0.001

We identified the correlation among pest density, soil physicochemical properties, soil enzyme activities, and bacterial and fungal alpha diversity in the three regions with *M. verticalis* larvae feeding. In the healthy aerobic rice rhizosphere soils (Fig. 7A), total N, total P, available P, and available K negatively correlated with soil enzyme activities. Bacterial number, bacterial Shannon index, and fungal Shannon index significantly positively correlated with soil physicochemical properties, whereas they significantly negatively correlated with phosphatase, β-glucosidase, and acid invertase. Fungal number significantly positively correlated with urease, β-glucosidase, polyphenol oxidase, and acid invertase. In the *M. verticalis* larvae-feeding aerobic rice rhizosphere soils (Fig. 7B), pest density significantly positively correlated with pH, organic matter, total P, catalase, and bacterial number and Shannon index but significantly negatively with available P, urease, phosphatase, β-glucosidase, and acid invertase. *M. verticalis* larvae-feeding changed many correlations, including reversed the correlation of organic matter and available P, total K and urease, and available P and fungal Shannon index, disrupted the correlation of pH with organic matter, available P, and fungal Shannon index, organic matter with total P, total N, available K, phosphatase, and et al., and generated new correlation of organic matter and total K, total P and fungal number, and acid invertase and bacterial number. Interestingly, *M. verticalis* larvae-feeding greatly reduced the correlation of fungi with soil physicochemical properties, soil enzyme activities, and bacterial community.

### Relationships between *M. verticalis* larvae density and aerobic rice rhizosphere soil microbial composition

**Fig. 8:**
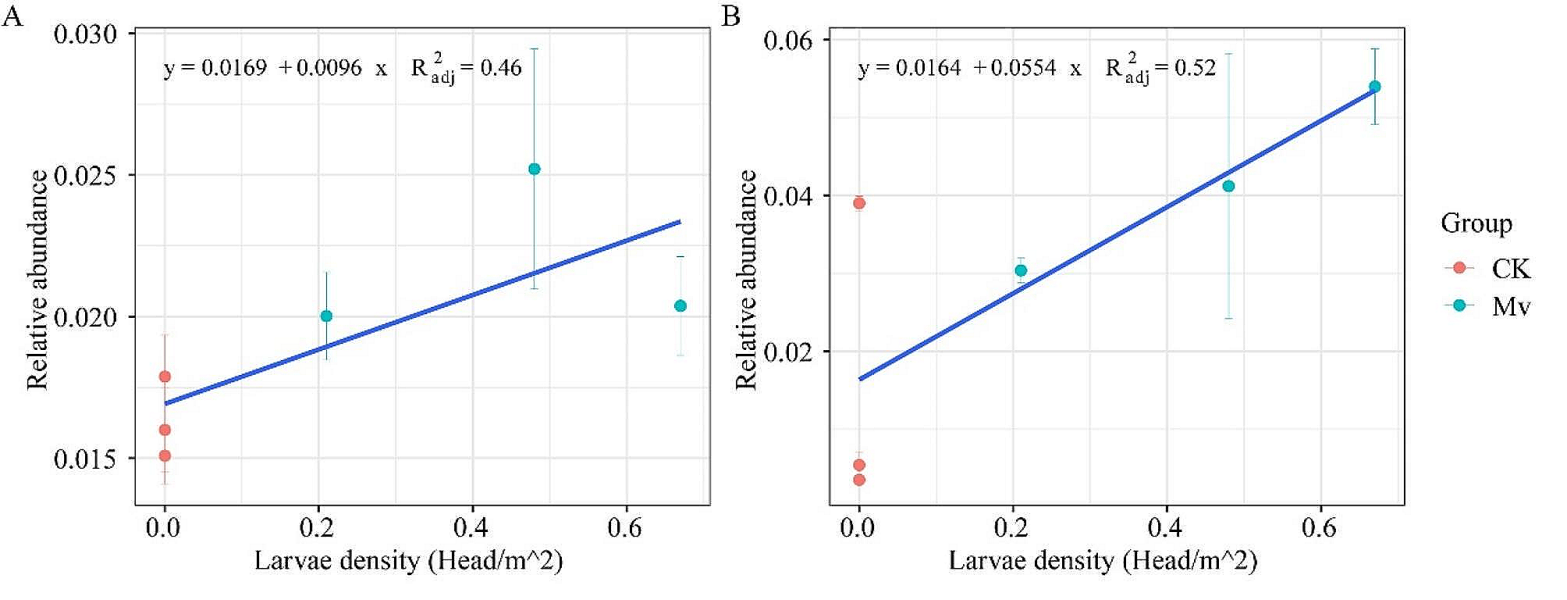
Relationships between *M. verticalis* larvae density and aerobic rice rhizosphere soil microbial composition. Relationships between *M. verticalis* larvae density and the relative abundance of *Bradyrhizobium* (**A**) and *Talaromyces* (**B**) genera. CK, healthy aerobic rice; Mv, *M. verticalis* larvae-feeding aerobic rice

The *Bradyrhizobium* and *Talaromyces* were the genera with the greatest difference between bacteria and fungi, respectively, after *M. verticalis* larvae-feeding. Therefore, we illustrated the relationships the relative abundance of *Bradyrhizobium* and *Talaromyces* and larvae density by using linear fitting. The fitting line in Fig. 8A showed that the relative abundance of *Bradyrhizobium* gradually increases with the elevation of the larvae density. The R^2^ of the fitting was 0.46, indicating a moderate correlation. The relative abundance of *Talaromyces* increases with the increases in larvae density (Fig. 8B). The high R^2^ of 0.52 suggested that the *Talaromyces* genus had a good correlation with larvae density.

## Discussion

Underground insects are closely related to soil’s physicochemical properties [35–37]. In this study, *M. verticalis* larvae feeding significantly changed soil pH and available K content in the three studied regions. The content of organic matter, total N, and available P were remarkably changed between two regions. Waste cricket chitin (*Acheta domesticus*) reduced the germination of *Lepidium sativum* and stimulated root elongation [36], which was closely related to soil physicochemical properties [38], suggesting that underground insects mediated changes in soil physicochemical properties that are critical for regulating plant’s growth and development. Furthermore, insect abundance of *Oulema* spp. larvae were negatively correlated with dehydrogenases, catalase, and peroxidases in soil [39]. Accumulating evidence shows that the expression of host-specific genes had a regulatory effect on soil enzyme activity [39–41]. Our results also showed that *M. verticalis* larvae density negatively correlated with soil enzyme (urease, phosphatase, β-glucosidase, and acid invertase) activities, suggesting that *M. verticalis* larvae-feeding stress mediated soil enzyme activities by regulating host‐specific genes expression.

Biotic and abiotic stresses can sculpt rhizosphere microbiota assembly and modulate the growth of root commensals that bolster stress tolerance [42]. Previous studies have shown that soil bacterial networks were less stable under biotic and abiotic stress than fungal networks [43–45]. In this study, we observed that the change of bacterial community was greater than fungal community with *M. verticalis* larvae-feeding stress, but *M. verticalis* larvae-feeding maintained the correlation of bacterial community with soil physicochemical properties and enzyme activities, suggesting that aerobic rice adapted to *M. verticalis* larvae-feeding stress by changing bacterial community. Increasingly more studies suggest that plant-mediated timely reshaping of the microbiota could also confer benefits in responding to certain biotic and abiotic stresses [46]. We observed that *M. verticalis* larvae-feeding significantly enriched *Bradyrhizobium* (bacterial genus) and *Talaromyces* (fungal genus), and the relative abundance of *Bradyrhizobium* and *Talaromyces* gradually increased with the elevation of the larvae density. Notably, *Bradyrhizobium* is one of the most cosmopolitan and diverse bacterial group modulating a variety of host legumes, which can promote growth, increase yield, and improve abiotic stresses resistance [47, 48] in various plants, including rice [49] peanuts [50], and maize [51]. In addition, *Talaromyces* can promote plant growth and inhibit pathogenic fungi and insects [52–54], such as *Talaromyces apiculatus* reduced linear mycelial growth of *Ganoderma boninense*, elevated area and bole girth of oil-palm seedlings, and increased shoot and root biomass, and nutrient contents in seedlings [52]. Combined with the results of this study, we speculate that aerobic rice may adapt to *M. verticalis* larvae-feeding stress by recruiting beneficial microorganisms.

Our experimental site is in Puer, Yunnan Province, China, where there is rich biodiversity [55, 56]. *M. verticalis* larvae-feeding will enrich many functional microorganisms compared to other places. Such as white grub (*H. parallela*) larvae infestation decreased the genus of *Rhodoplanes* and *Ramlibacter* [18], but they were increased in our study. Notably, *M. verticalis* larvae-feeding increased the genus of *Bradyrhizobium* and *Talaromyces* in this study, which were functional microorganisms that were beneficial for plants to adapt to stress [47, 48, 52–54], suggesting that they can be made into biological agents for use. However, specific species had not been isolated and identified.

We observed that seven featured genera were identified in the healthy aerobic rice rhizosphere, including the PGPR *Mucilaginibacter* [57] and *Dyella* [58] genera. *M. verticalis* larvae-feeding aerobic rice rhizosphere bacteria featured 22 genera, including the PGPR *Terrimonas* [59] and *Bradyrhizobium* [60, 61] genera. PGPR can successfully colonize insects and use them as a means of dispersal to the rhizosphere of new host plants [62], suggesting that *M. verticalis* larvae may have brought over these 22 featured genera. *M. verticalis* larvae feeding significantly depleted the enrichment of the pathway of metabolism of carbohydrate metabolism at KEGG level 2. Carbohydrate metabolism is the dominant bacterial function in halophytes rhizosphere bacteria and light-exposure rhizosphere bacteria [63, 64]. Notably, rhizosphere bacteria were significantly affected by soil physicochemical characteristics [65, 66]. In our study, bacterial number and Shannon index correlated significantly positively with pH, total N, total P, available K, and NH4+-N. These results indicate that 22 featured bacterial genera from *M. verticalis* larvae-feeding aerobic rice rhizosphere are strongly associated with soil physicochemical properties.

Many rhizosphere fungi belong to the arbuscular mycorrhizal fungi (AMF) and dark septate fungi (DSE). By forming symbiotic relationships with most plants, AMF and DSE can improve salt tolerance, disease resistance, and water stress [67–70]. In this study, eight featured genera were identified in the healthy aerobic rice rhizosphere, including the AMF *Penicillium* genus [71]. *M. verticalis* larvae-feeding aerobic rice rhizosphere bacteria featured 13 genera, including one DSE *Exophiala* genus [72], three AMF *Acremonium*, *Mortierella*, and *Lecythophora* genera [71, 73], and P solubilization fungi *Talaromyces* genus [74]. Notably, we found that *M. verticalis* larvae feeding significantly decreased the abundance of fungal parasite–wood saprotroph and fungal parasite, possibly because insect ectomicrobiomes produce antimicrobial peptides or antibiotic compounds [75]. The reduction in such fungi reduces the degradation of soil organisms, leading to a decrease in the nutrients obtained by aerobic rice plants from the soil. *M. verticalis* larvae feeding significantly increased the abundance of plant pathogen–wood saprotroph and animal pathogen–undefined saprotroph. Their increase may be carried out by plant pathogens and animal pathogens via *M. verticalis* larvae [76, 77]. Here, we found that rhizosphere fungal number and Shannon index strongly correlated with soil physicochemical properties and soil enzyme activities. Rhizosphere fungal community structures were primarily shaped by the total N, available N, and available P of soil [78], which increased the activity of chitinase, leucine-aminopeptidase, acid phosphatase, and β-glucosidase [79]. However, we did not determine if the high correlation between fungal activity and soil enzyme activity is due to fungal secretion. Finally, the relationship between soil physicochemical properties, soil enzyme activities, and rhizosphere fungi should be investigated in the future.

However, this study also raises some issues that must be clarified in future investigations. Firstly, the isolation and identification of *Bradyrhizobium* and *Talaromyces* species is important for subsequent functional research. Further studies would need to focus on the application of key functional microorganisms. Secondly, this study doesn’t have a good way to eliminate the potential confounding factors, such as environmental variations, other pest interactions, and agronomic practices, and a long-term testing experiment was necessary. Finally, the mechanism by which *M. verticalis* larvae feeding drives changes in aerobic rice rhizosphere soil microbial communities is still unclear.

## Conclusions

This study demonstrates that *M. verticalis* larvae feeding can influence soil physicochemical properties and soil enzyme activities, which also changed the rhizosphere microbial communities and increased the featured microbial genera of the aerobic rice rhizosphere. In addition, the pathway of the metabolism of terpenoids and polyketides, carbohydrate metabolism, and immune system at KEGG level 2 and the pathway of galactose metabolism and amino sugar and nucleotide sugar metabolism at KEGG level 3 were significantly increased after *M. verticalis* larvae feeding. Fungal parasite–wood saprotroph and fungal parasite were significantly decreased after *M. verticalis* larvae feeding, and plant pathogen–wood saprotroph and animal pathogen–undefined saprotroph were increased. Pest density was most closely associated with the relative abundance of *Bradyrhizobium* and *Talaromyces*. These results provide new insights for understanding the adaptation of aerobic rice to *M. verticalis* larvae feeding via regulating the rhizosphere environment.

### Electronic supplementary material

Below is the link to the electronic supplementary material.

Supplementary Material 1

Supplementary Material 2

Supplementary Material 3

Supplementary Material 4

Supplementary Material 5

Supplementary Material 6

Supplementary Material 7

Supplementary Material 8

Supplementary Material 9

## Data Availability

Rhizosphere bacteria and fungi raw data have been deposited in the NCBI Sequence Read Archive (https://submit.ncbi.nlm.nih.gov/subs/sra/) under the accession number of PRJNA1052402 and PRJNA1053271, respectively.

## Abbreviations

PGPR: plant-growth-promoting rhizobacteria
CK: healthy aerobic rice
Mv: *M. verticalis larvae-feeding aerobic rice*
HGM: Haguoma
HZB: Haoziba
NG: Nuoguo
CFU: colony-forming units
PCR: polymerase chain reaction
OUT: operational taxonomic unit
PCA: principal component analysis
LDA: linear discriminant analysis
KEGG: Kyoto Encyclopedia of Genes and Genomes
AMF: arbuscular mycorrhizal fungi
DSE: dark septate fungi
