# Supplementary material for: The effect of white grub (Maladera Verticalis) larvae feeding on rhizosphere microbial characterization of aerobic rice (Oryza sativa L.) in Puer City, Yunnan Province, China
Source: BMC Microbiol. 2024 Apr 15;24:123. doi: 10.1186/s12866-024-03265-w (PMC11017655; doi:10.1186/s12866-024-03265-w)
Supplement: Supplementary file 1 — Supplementary Material 1 [file 12866_2024_3265_MOESM1_ESM.pdf]

The effect of white grub (*Maladera verticalis*) larvae feeding on rhizosphere microbial characterization of aerobic rice (*Oryza sativa* L.) in Puer City, Yunnan Province, China

Guang Wang[1†], Zhengfei Li[1†], Baoyun Yang[1], Huquan Yang[1], Yujie Zhang[1], Qingping Zeng[1], Chaojianping Yan[1], Yanyan He[1, 2], Yuejin Peng[1], Wenqian Wang[1], Bin Chen[1], and Guangzu Du[1*]

[1]State Key Laboratory of Conservation and Utilization of Biological Resources of Yunnan, College of Plant Protection, Yunnan Agricultural University, Kunming 650201, China

[2]School of Agriculture, Yunnan University, Kunming 650500, China

[*]Correspondence: Correspondence: duguangzu1986@163.com (G.D.)

[†]These authors contributed equally to this work

Supplementary Figure 1: OTU rarefaction curve (PDF).

Supplementary Figure 2: PCA showed separation patterns caused by M. verticalis larvae feeding in aerobic rice rhizosphere bacteria (PDF).

Supplementary Figure 3: PCA showed separation patterns caused by M. verticalis larvae feeding in aerobic rice rhizosphere fungi (PDF).

Supplementary Figure 4: Venn diagram showing the number of common and unique featured genera from the three regions (PDF).

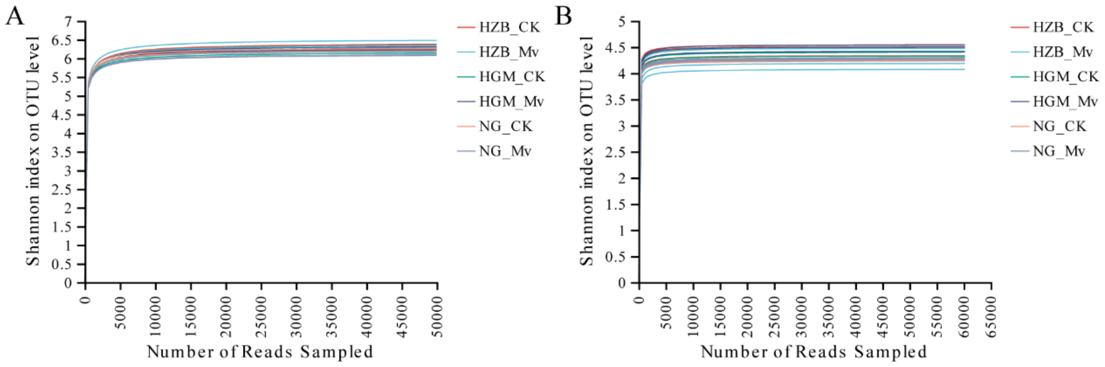

Figure S1. OTU rarefaction curve. Rarefaction curve derived from 16S rDNA (**A**) and ITS (**B**) sequencing.

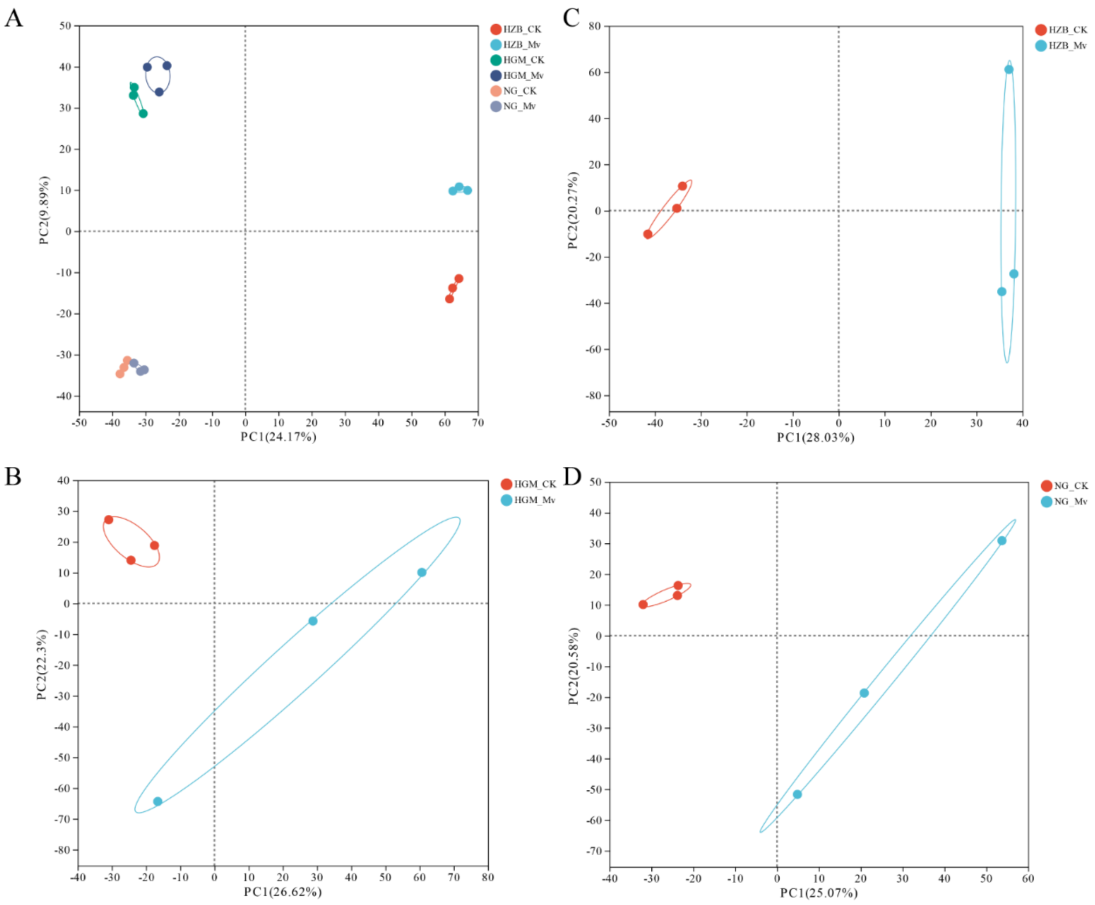

Figure S2. PCA showed separation patterns by *M. verticalis* larvae feeding in aerobic rice rhizosphere bacteria. (**A**) The PCA plots of beta diversity between healthy and *M. verticalis* larvae-feeding aerobic rice rhizosphere soil in the three regions. The PCA plots of beta diversity between healthy and *M. verticalis* larvae-feeding aerobic rice rhizosphere soil in the HGM (**B**), HZB (**C**), and NG (**D**). PCA, principal component analysis; CK, healthy aerobic rice; Mv, *M. verticalis* larvae-feeding aerobic rice; HGM, Haguoma; HZB, Haoziba, NG, Nuoguo.

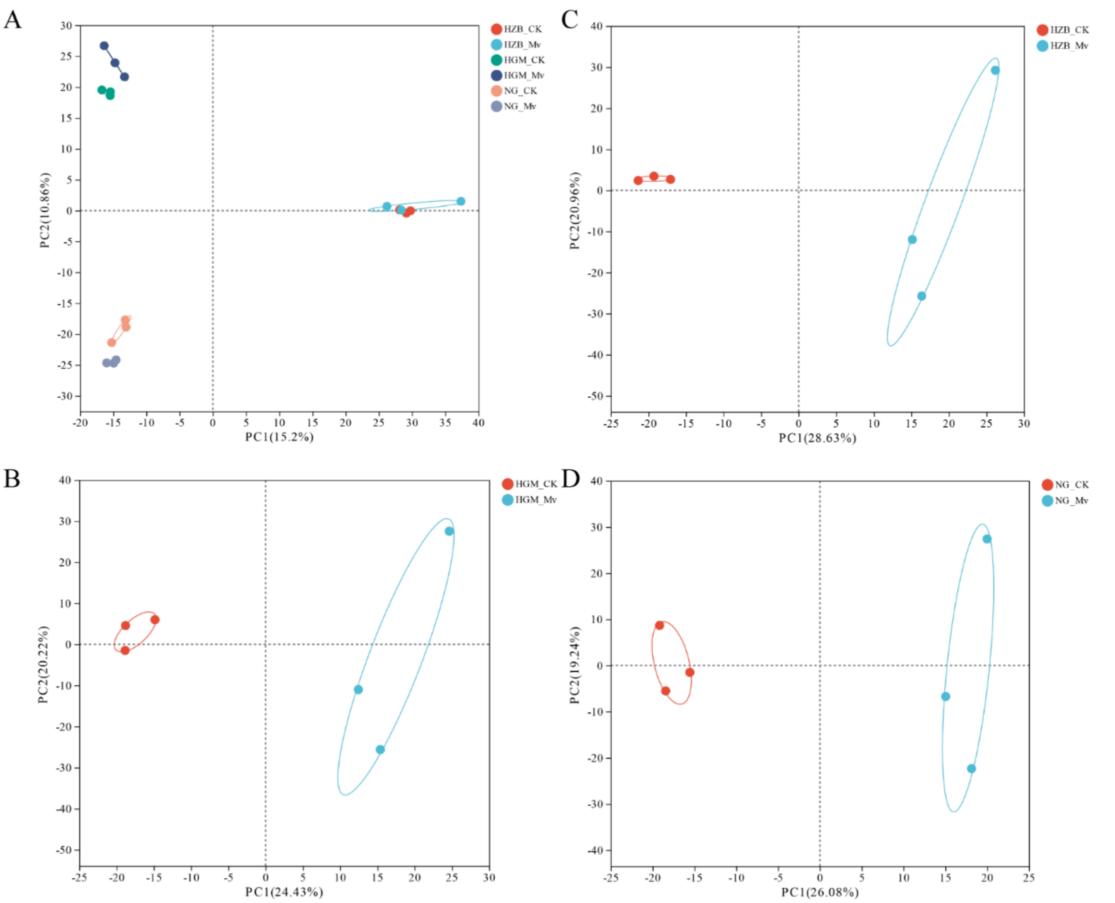

Figure S3. PCA showed separation patterns by *M. verticalis* larvae-feeding in aerobic rice rhizosphere fungi. (**A**) The PCA plots of beta diversity between healthy and *M. verticalis* larvae-feeding aerobic rice rhizosphere soil in the three regions. The PCA plots of beta diversity between healthy and *M. verticalis* larvae-feeding aerobic rice rhizosphere soil in the HGM (**B**), HZB (**C**), and NG (**D**). PCA, principal component analysis; CK, healthy aerobic rice; Mv, *M. verticalis* larvae-feeding aerobic rice; HGM, Haguoma; HZB, Haoziba, NG, Nuoguo.

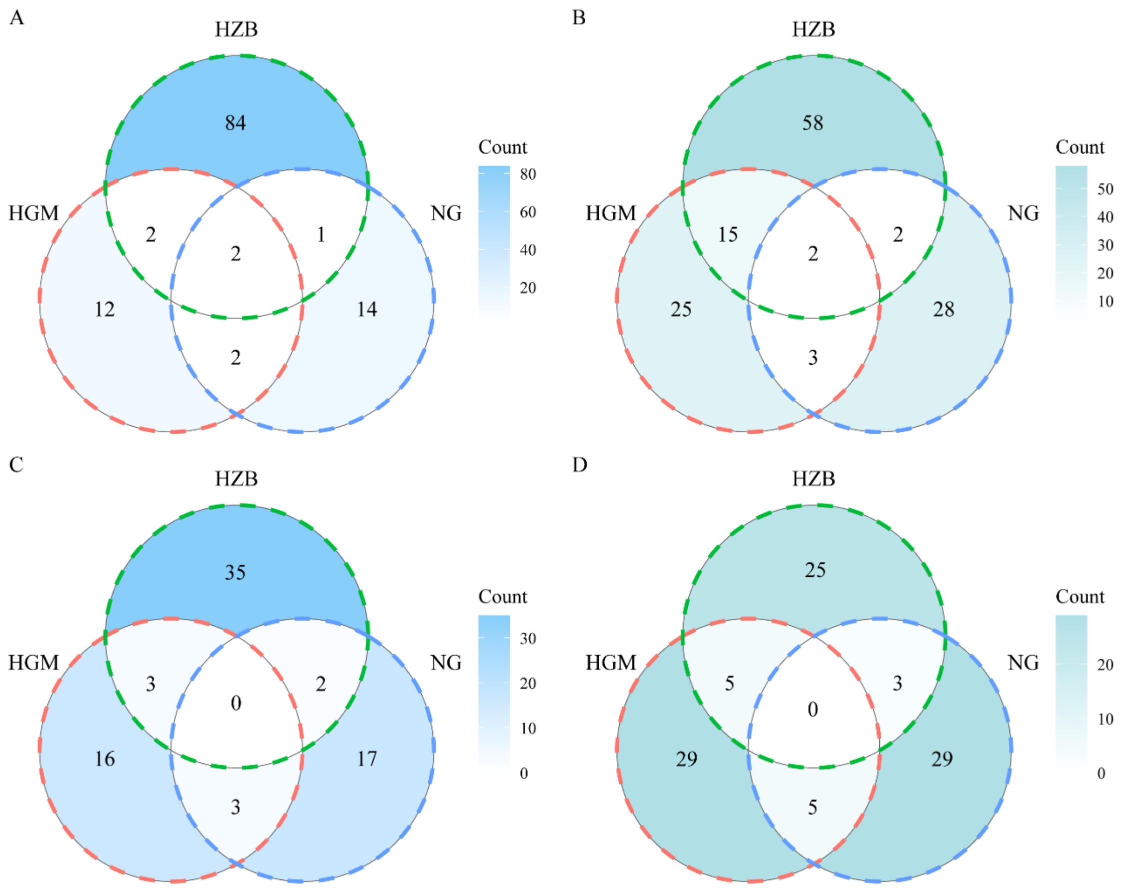

Figure S4. Venn diagram showing the number of common and unique featured genera from regions. (**A**) Venn diagram showing in the healthy aerobic rice rhizosphere bacteria. (**B**) Venn diagram showing in the *M. verticalis* larvae-feeding aerobic rice rhizosphere bacteria. (**C**) Venn diagram showing in the healthy aerobic rice rhizosphere fungi. (**D**) Venn diagram showing in the *M. verticalis* larvae-feeding aerobic rice rhizosphere fungi. HGM, Haguoma; HZB, Haoziba, NG, Nuoguo.

Supplementary Table 1: Statistics of effective sequencing data of bacteria and fungi (WROD).

Supplementary Table 2: The relative abundance of rhizosphere bacteria at genus level (XLSX).

Supplementary Table 3: The relative abundance of rhizosphere fungi at genus level (XLSX).

Supplementary Table 4: The parameter of aerobic rice rhizosphere soil microbial community (XLSX).

Supplementary Table 5: The relative abundance of phyla in aerobic rice rhizosphere soil microbial community (XLSX).

Supplementary Table 6: Statistical differences in predicated bacterial functional characteristics between CK and Mv rhizosphere soil at KEGG level 2 (XLSX).

Supplementary Table 7: Statistical differences in predicated bacterial functional characteristics between CK and Mv rhizosphere soils at KEGG level 3 (XLSX).

Supplementary Table 8: Statistical differences in predicated fungal functional characteristics between CK and Mv rhizosphere soils (XLSX).
