# Supplementary material for: The effect of white grub (Maladera Verticalis) larvae feeding on rhizosphere microbial characterization of aerobic rice (Oryza sativa L.) in Puer City, Yunnan Province, China
Source: BMC Microbiol. 2024 Apr 15;24:123. doi: 10.1186/s12866-024-03265-w (PMC11017655; doi:10.1186/s12866-024-03265-w)
Supplement: Supplementary file 8 — Supplementary Material 8 [file 12866_2024_3265_MOESM8_ESM.docx]

Table S1. Statistics of effective sequencing data of bacteria and fungi.

| Samples | Bacteria | |  | Fungi | |
| --- | --- | --- | --- | --- | --- |
|  | OTU numbers | Coverage |  | OTU numbers | Coverage |
| HGM_CK | 3254.33 ± 13.91 | 0.9861 ± 0.0007 |  | 726.00 ± 21.46 | 0.9980 ± 0.0004 |
| HGM_Mv | 3225.33 ± 8.58 | 0.9851 ± 0.0003 |  | 756.00 ± 20.61 | 0.9981 ± 0.0004 |
| HZB_CK | 2651.00 ± 42.67 | 0.9834 ± 0.0000 |  | 705.00 ± 4.24 | 0.9979 ± 0.0001 |
| HZB_Mv | 2857.33 ± 41.48 | 0.9836 ± 0.0007 |  | 720.00 ± 32.44 | 0.9983 ± 0.0002 |
| NG_CK | 2432.33 ± 34.50 | 0.9885 ± 0.0003 |  | 672.33 ± 12.92 | 0.9980 ± 0.0001 |
| NG_Mv | 2522.67 ± 15.92 | 0.9870 ± 0.0005 |  | 724.33 ± 12.12 | 0.9978 ± 0.0001 |
